# Optical Properties of Perovskite‐Organic Multiple Quantum Wells

**DOI:** 10.1002/advs.202200379

**Published:** 2022-07-03

**Authors:** Tobias Antrack, Martin Kroll, Markas Sudzius, Changsoon Cho, Paulius Imbrasas, Miguel Albaladejo‐Siguan, Johannes Benduhn, Lena Merten, Alexander Hinderhofer, Frank Schreiber, Sebastian Reineke, Yana Vaynzof, Karl Leo

**Affiliations:** ^1^ Dresden Integrated Center for Applied Physics and Photonic Materials (IAPP) and Institute for Applied Physics Technische Universität Dresden Nöthnitzer Str. 61 01187 Dresden Germany; ^2^ Institut für Angewandte Physik Universität Tübingen Auf der Morgenstelle 10 72076 Tübingen Germany

**Keywords:** amplified spontaneous emission, confinement, luminescence, perovskite, quantum well, simulation, vacuum deposition, X‐ray reflectivity

## Abstract

A comprehensive study of the optical properties of CsPbBr_3_ perovskite multiple quantum wells (MQW) with organic barrier layers is presented. Quantum confinement is observed by a blue‐shift in absorption and emission spectra with decreasing well width and agrees well with simulations of the confinement energies. A large increase of emission intensity with thinner layers is observed, with a photoluminescence quantum yield up to 32 times higher than that of bulk layers. Amplified spontaneous emission (ASE) measurements show very low thresholds down to 7.3 µJ cm^−2^ for a perovskite thickness of 8.7 nm, significantly lower than previously observed for CsPbBr_3_ thin‐films. With their increased photoluminescence efficiency and low ASE thresholds, MQW structures with CsPbBr_3_ are excellent candidates for high‐efficiency perovskite‐based LEDs and lasers.

## Graphical abstract

CsPbBr_3_ perovskite/TPBi organic multiple quantum wells are reported to show charge carrier confinement that results in a massive increase of emission efficiency with stronger confinement. Waveguide formation allows to reach extremely low thresholds of amplified spontaneous emission, which is important for future laser applications.

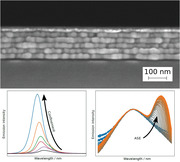

## 1 Introduction

Metal halide perovskite materials are direct‐bandgap semiconductors that have caught the attention of researchers in the field of optoelectronic devices over the last decade,^[^ ^1^ ^]^ due to their excellent properties such as long carrier lifetime,^[^ ^2^ ^]^ low defect trap density,^[^ ^3^ ^]^ and large absorption coefficient.^[^ ^4^ ^]^ Remarkable advances have led to high performance in both solar cells (power conversion efficiencies > 25%^[^ ^5^ ^]^) and light–emitting diodes (external quantum efficiency > 20% ^[^ ^6^, ^7^ ^]^) based on perovskite materials. Additionally, perovskites are of special interest for high‐fluence applications such as lasers due to their very high damage thresholds, narrow linewidth, and high optical gain.^[^ ^8^ ^]^ Since the bandgap can be tuned over the entire visible light spectrum by either modifying the composition^[^ ^9^ ^]^ or tailoring the dimensionality,^[^ ^10^ ^]^ perovskite‐based lasers are not only possible alternatives for already existing laser devices, but would rather open a full spectrum of new applications^[^ ^11^ ^]^ by filling the spectral gaps where common lasers cannot emit.

Most of the research in perovskite‐based light‐emitting applications has been based on perovskite materials fabricated by solution processing.^[^ ^12^ ^]^ However, perovskite thin films can also be manufactured by vacuum deposition, which has already shown to be more promising for photon amplification.^[^ ^13^ ^]^ Vacuum deposition is easily scalable, which paves the way for a more facile realization of industrial‐scale perovskite optoelectronic devices.^[^ ^14^ ^]^ Another important benefit of deposition by thermal evaporation is the possibility to fabricate complex multilayer structures with precise control of layer thicknesses down to sub‐nanometer scale.

When using an alternating heterostructure of materials possessing different bandgaps, multiple quantum wells (MQWs) can be created if the layers are sufficiently well‐defined.^[^ ^15^ ^]^ With a well width comparable to the exciton Bohr diameter, the charge carriers are confined and the energy levels become discrete with a step‐like density of states. The exciton Bohr diameter of bulk CsPbBr_3_ perovskite, as the one used in this study, is 7 nm.^[^ ^16^, ^17^ ^]^ In this small volume of space, the carrier concentration is very high and electron–hole recombination is enhanced, which enables a stronger population inversion.^[^ ^18^ ^]^ These advantages make MQWs a field of intense research, leading to the demonstration of superluminescent light‐emitting diodes, infrared photodetectors, and quantum cascade lasers with very high efficiencies.^[^ ^18^, ^19^, ^20^, ^21^ ^]^

Combining the advantages of perovskites with the improvements that can be achieved by their integration into MQW structures is a promising method to approach the goal of electrically driven perovskite‐based lasers. As steps toward that goal, electrical pumping of perovskite diode structures^[^ ^22^ ^]^ and amplified spontaneous emission (ASE) of films^[^ ^13^ ^]^ have been shown. MQW structures made of CsPbBr_3_ and 1,3,5‐tris(2‐*N*‐phenylbenzimidazolyl) benzene (TPBi) layers were already presented in ref. [15] where the authors have shown quantum confinement by blue shifts of emission and absorption spectra with decreasing well thickness.

**Figure 1:**
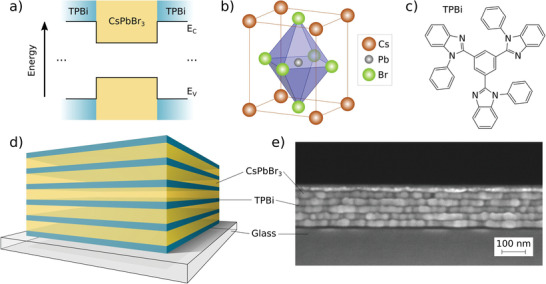
a) Schematic sketch of the band structure of a single quantum well. The structure is repeated five times to create the MQWs. b) Unit cell of orthorhombic CsPbBr_3_ perovskite and c) chemical structure of TPBi. d) General structure of the samples: The CsPbBr_3_ perovskite thickness was varied from 3–20 nm between samples and the TPBi thickness was kept constant at 5 nm. e) Cross‐section SEM of an MQW 20 sample with five 20 nm‐thick perovskite layers (bright) confined in 5 nm‐thick TPBi layers (dark) on a glass substrate. The precise determination of thicknesses was conducted via small‐angle X‐ray reflectivity (XRR, see Supporting Information for more details).

Here, we present a comprehensive study of type‐I multi‐quantum well structures and their potential for use in perovskite‐based light‐emitting or even lasing devices. For this purpose, similar to the previous report by Lee et al.,^[^ ^15^ ^]^ TPBi was used as an organic blocking layer between CsPbBr_3_ perovskite layers to realize quantum wells for both electrons and holes simultaneously (**Figure** **1**a–d). Both materials were sequentially deposited on glass substrates via thermal evaporation. We demonstrate that the MQW structure has optical properties far superior to bulk films.

## 2 Results and Discussion

To investigate the effect of the perovskite layer thickness on the MQW properties, five different sample structures were produced by vacuum deposition. In each structure, five perovskite layers of varying thicknesses (3, 5, 7, 10, and 20 nm) were sandwiched between blocking layers (TPBi) with a thickness of 5 nm. The general MQW structure, and a scanning electron microscopy (SEM) cross‐section of the sample with a perovskite thickness of 20 nm are shown in Figure 1d,e. Additionally, a thin film sample consisting of a 50 nm‐thick perovskite layer enclosed between two 5 nm‐thick TPBi layers was fabricated as reference. In the following, the MQW samples are labeled as MQW 3, 5, 7 .. and the bulk sample is referred to as bulk 50.

### 2.1 Structural Characterization of the MQWs

**Figure 2:**
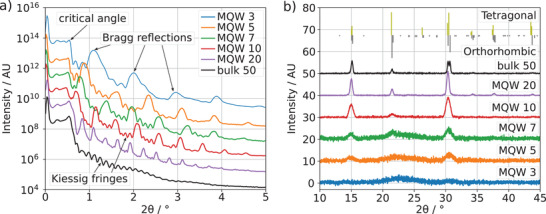
a) X‐ray reflectivity patterns of different layer thicknesses. The total reflection edge is at 2 Θ ≈ 0.5 °. For higher angles, characteristic Bragg peaks due to reflections at layer interfaces and thickness oscillations (Kiessig fringes) are clearly visible. b) X‐ray diffraction data of different layer thicknesses and the reference for orthorhombic and tetragonal CsPbBr_3_.^[^ ^24^ ^]^

SEM cross‐section of the MQW 20 sample (Figure 1e) shows that compact and pinhole‐free perovskite films are formed for these layer thicknesses, which is crucial for the observation of quantum confinement effects. Similar formation of well distinguishable layers for thinner samples was confirmed by small‐angle X‐ray reflectivity (XRR) measurements. In these measurements, homogeneous films with small surface roughness result in a characteristic series of Bragg reflections corresponding to the periodic layer thicknesses in the MQW structures. In **Figure** **2**a, clear Bragg reflections are observed, which confirm the formation of clearly distinguishable layers. In addition, we find thickness oscillations (Kiessig fringes) corresponding to the thickness of the entire MQW structure.^[^ ^23^ ^]^ The experimental results were fitted assuming an electron density profile where all layers of the same type have the same thickness, and the r esults are summarized in Table S1, Supporting Information. We find excellent agreement between the nominal film thicknesses and those extracted from the XRR measurements.

The deposition of ultrathin perovskite layers by thermal evaporation raises questions regarding their crystalline quality. To investigate this, the MQW structures and the bulk reference samples were characterized by X‐ray diffraction (XRD). The XRD diffractograms shown in Figure 2b confirm the successful formation of perovskite layers with a tetragonal structure for the layered structures and orthorhombic for the bulk.^[^ ^24^ ^]^ While no clear reflexes were observed for the MQW 3 structure, we believe that this is caused by the very small amount of perovskite materials in these samples that does not lead to measurable X‐ray diffraction. Please note that between 20° and 25°, the amorphous background of the glass substrate becomes noticeable for the thinner perovskite layers.

### 2.2 Optical Characterization of the MQWs

**Figure 3:**
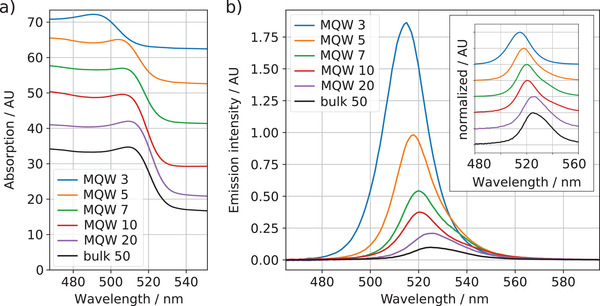
Optical properties of the investigated samples. a) Absorption spectra for different layer thicknesses. b) Photoluminescence spectra of MQWs when excited by a 405 nm CW laser, the inset shows the normalized spectra (measured in an integrating sphere, see Experimental Section for more details).

The optical properties of materials confined in a quantum well structure depend on the width of the well, leading to a blue‐shift in absorption for narrower quantum wells.^[^ ^25^ ^]^ **Figure** **3**a shows the measured optical absorption spectra, clearly shifting toward shorter wavelengths (e.g., higher energies) for smaller perovskite thicknesses. Additionally, the same trend can be observed in the emission spectra (Figure 3b). These shifts are typical for quantum well structures due to the higher confinement energies at smaller well sizes.

In contrast to our observations, Lee et al., measured much larger energy shifts for the same perovskite thicknesses in single quantum well structures.^[^ ^15^, ^26^ ^]^ Assuming variations in the perovskite thickness, charge carriers are expected to accumulate at positions with slightly higher well thickness so they can minimize their energy. The relatively high values of surface roughness obtained via XRR (Table S1, Supporting Information) are in agreement with this effect. Additionally, as Lee et al.,^[^ ^15^ ^]^ has already shown for a barrier thickness of 7 nm, tunneling effects cannot be excluded. Charge carriers are hence able to tunnel through the TPBi barrier and accumulate at the thickest CsPbBr_3_ well. If the formation of a closed layer was not successful, isolated grains of perovskite could exist embedded in TPBi that would behave like a quantum dot due to the confinement in all dimensions. CsPbBr_3_ quantum dots with similar diameters as the thickness of the thin layers in our work show a significantly larger blue shift in emission and absorption spectra than observed for our quantum wells.^[^ ^27^ ^]^ Therefore, we additionally assume that we have a successful formation of closed perovskite layers. 2D confinement in lead halide perovskites has also been shown in Ruddlesden–Popper perovskites, where confinement energies of 500 meV^[^ ^28^, ^29^ ^]^ are reached, which is about one order of magnitude stronger than our observations. This strong confinement is achieved by creating well thicknesses down to one unit cell.

### 2.3 Confinement Simulation

To verify the observed band gap shifts due to confinement, the MQW structures were simulated by numerically solving the Schrödinger equation employing the method of finite differences. This method creates discrete approximations that can be solved using linear equations, and by that preserves important properties of the underlying continuum equation. As sketched in Figure S6, Supporting Information, a simple 1D step‐like potential is assumed which represents the potential curve of the conduction band for electrons or the valence band for holes. Well heights of 0.41 eV in the conduction band and 0.55 eV in the valence band were assumed,^[^ ^26^ ^]^ and the effective masses in the well were set to 0.15 *m*_e_ for electrons and 0.14 *m*_e_ for holes.^[^ ^30^ ^]^

Solving the eigenvalue problem results in multiple eigenenergies for each well thickness. Only the ground states are used, since charge carriers elevated into higher energy levels are expected to relax to the ground state very fast. The simulation was performed for electrons in the conduction band and for holes in the valence band, and the obtained minimum eigenenergies were added to get the total confinement energies.

**Figure 4:**
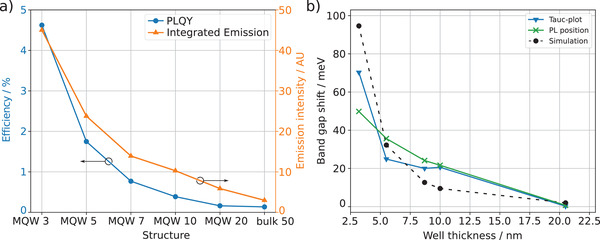
a) Integrated emission intensity and Photoluminescence Quantum Yield (PLQY). For the MQW 3, the integrated emission intensity is more than 15 times larger than that of the bulk and the PLQY is increased by 35 times. b) Band gap shift depending on the well thickness (obtained by XRR measurements) of the MQWs compared to the bulk energies. The absorption spectra are analyzed via the Tauc‐plot method, the peak position of the photoluminescence emission spectrum is used, and the simulation results are determined by finding the eigenenergies within the quantum wells.

Since the energy eigenstates heavily depend on the effective masses in well and barrier and the effective masses for TPBi are unknown, the simulation is conducted by fitting the obtained band gap shifts to the measured values depending on the barrier effective masses. The best‐fitting value is reached by using the least‐square method between the simulation results and both the data from the Tauc‐plot and from the spectral emission position. By that, effective masses of 0.46 *m*_e_ for electrons and 2.07 *m*_e_ for holes were obtained, which are within the range of other organic semiconductors.^[^ ^31^, ^32^, ^33^ ^]^ The resulting band gap shifts (**Figure** **4**b) agree well with the gap shifts obtained from the emission spectra. By that, successful quantum confinement is additionally confirmed. Conducting the simulation for single quantum wells with the beforehand obtained effective mass of TPBi results in 1.6% higher energy levels. This small difference is explained by the presence of adjacent quantum wells in the MQW structure, which allows coupling between the wave functions of the individual quantum wells.

### 2.4 Photoluminescence Quantum Yield

To our knowledge, there is currently no report about emission intensity of CsPbBr_3_/TPBi MQW structures. Figure 3b shows that the emission intensity is significantly increasing for smaller well thickness. Integrating the emission signals results in an emission intensity 15 times higher for the MQW 3 compared to the bulk (Figure 4a). As a result of lower absorbance of the thinner samples, MQW structures with lower well width exhibit a much higher photoluminescence quantum yield (PLQY) than thicker wells. The efficiency increases from 0.13% for the bulk to 4.62% for the MQW 3, which is an increase by a factor of 35.

Excitons are known to dissociate in bulk CsPbBr_3_.^[^ ^34^ ^]^ By spatially confining the excitons using quantum wells, the probability of dissociation can be lowered, thus, the radiative recombination is becoming more likely which results in the observed increase of emission intensity. The enhanced overlap of the electron and hole wave functions for thin wells additionally increases the probability for radiative recombination. Furthermore, the high surface roughnesses observed in XRR increases the outcoupling efficiency.

Since PLQY represents the ratio of emitted photons per electronic excitation, it is a crucial factor for designing efficient LEDs or lasers.^[^ ^35^ ^]^ These new findings show that MQW structures can pave the way to high‐efficiency perovskite‐based LEDs.

As shown in Section 2.6, samples with a total structure thickness above 60 nm exhibit waveguide formation in the spectral range of emission. If waveguiding (and therefore higher absorption within the layer) in thicker structures would be the main reason for the observed increase in emission intensity of thinner structures, the emission intensity of bulk 50 would be in the range of MQW 5 and MQW 7 (maybe even higher since it contains more perovskite material, see Table S1, Supporting Information). However, since the bulk 50 shows the lowest PLQY, it is clear that waveguiding only plays a minor role in influencing PLQY. For a precise determination, detailed simulations would be necessary.

### 2.5 Decay Properties

For luminescence decay trace measurements, a picosecond time correlated single photon counting technique (TCSPC) is used. The decay traces are fitted using a model assuming two exponential decay components and a power‐law decay (see Supporting Information for more details). There is a clear trend observed toward longer decay times for thinner perovskite layers, and MQW 3 exhibits a power‐law decay constant of 1.5 ns, which is significantly higher than for the other samples, where it stays below 0.5 ns (see Table S2, Supporting Information). This result suggests that the effective charge carrier mobility, and therefore the recombination rate of free charge carriers, is lowered due to a higher probability of scattering in the thin quantum well. More experiments are needed to unveil the underlying process resulting in different power‐law decay constants. For the exponential decay times, there is a clear difference observed between the MQWs and the bulk structure: the first exhibit decay times around an average of 0.35 ns while in the latter excitons decay much faster with 0.19 ns. For a full understanding of the decay characteristics, further studies including temperature and density dependencies are needed.

### 2.6 Amplified Spontaneous Emission

In optical material systems where a single‐pass gain is sufficiently large, the spontaneously emitted photons may undergo amplification even if the system lacks an efficient mechanism for positive optical feedback. This process of photon amplification can be identified unambiguously from the measurements of the output intensity as a function of optical pump power (input–output characteristics, IO). Assuming that the stimulated emission threshold is reached, the IO shows an S‐shaped dependence due to a non‐linear increase from spontaneous emission to the ASE regime, which is typical for laser devices.^[^ ^36^, ^37^ ^]^ Here, we investigated ASE properties of the CsPbBr_3_/TPBi MQW structures, measuring and using the ASE threshold as a material parameter, which characterizes perovskite‐based MQW as a gain medium and reveals its potential for lasing applications.

**Figure 5:**
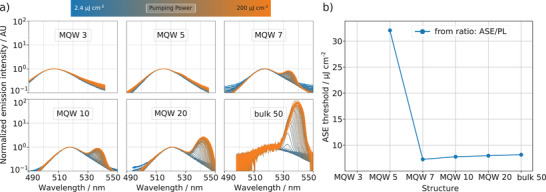
a) Normalized ASE spectra in various MQW systems, showing the phenomenon of spectral narrowing in the transitional region from spontaneous to stimulated regime and b) determined ASE thresholds for these structures.

**Figure** **5**a shows experimentally measured normalized ASE spectra in various perovskite‐based MQW systems. With increasing pump intensity, a narrow second spectral peak arises on the low‐energy shoulder of the PL spectrum for the structures with total thicknesses exceeding 60 ~nm (see Table S1, Supporting Information). This spectral narrowing phenomenon is typical for the ASE processes in the transitional region from spontaneous to stimulated regime. The spectral red‐shift of the ASE peak with respect to the PL spectrum is caused by a relatively small Stokes shift and thus the interplay between absorption and emission bands, which overlap substantially in the energy domain (see also Figure 3). This leads to a significant spectral shift of the ASE peak to the red side of the spectrum, as the optimal conditions (i.e., the balance between gain, loss, and optical density of states) for the ASE to develop happens on the very tail of the absorption band, where optical losses vanish rapidly. The ASE does not develop in the MQW 3 structure as its overall thickness barely exceeds 50 nm, whereas in ref. [13] it was determined that a minimum thickness of 60 nm is necessary to allow waveguide formation in a very similar structure. Therefore, the thinnest structure is insufficient to support any waveguided modes in the spectral region where the gain peak of the perovskite material evolves. For larger thicknesses of the structure, the optical mode confinement starts to improve substantially, which in turn causes a reduction of the ASE threshold.^[^ ^38^ ^]^

Figure 5b summarizes ASE thresholds for these structures. The thresholds have been determined from the corresponding IO dependencies from Figure 5a). To determine the ASE threshold, the ASE and PL emission has to be separated, and the pumping‐dependent ratio *R*_ASE/PL_ of the spectral intensity of the ASE peak to the PL peak has to be examined. More details about the used method can be found in Supporting Information. Here, we report ASE thresholds as low as 7.3, 7.8, 8.0, and 8.2 µJ cm^−2^ for the MQWs 7, 10, 20, and the bulk, respectively. These very low ASE thresholds are unprecedented for CsPbBr_3_ thin film structures inspite of high layer roughnesses observed in XRR, which gives rise to a parasitic light scattering and prevents formation of well confined waveguide modes. In previous works, much higher ASE thresholds of 35 µJ cm^−2^ for vacuum deposited CsPbBr_3_ films with thicknesses of 35 nm and above,^[^ ^13^ ^]^ and 2.55 mJ cm^−2^ for solution processed CsPbBr_3_^[^ ^39^ ^]^ are reported. Using CsPbBr_3_ quantum dots, thresholds as low as 12~µJ cm^−2^ are reached through two‐photon absorption processes.^[^ ^40^ ^]^

A clear trend of increasing threshold intensity with increasing layer thickness is observed beginning at MQW 7 (Figure 5b). Since mode confinement is expected to monotonically increase with layer thickness, the ASE threshold should decrease.^[^ ^38^ ^]^ To explain the observed behavior, the lower thickness of total perovskite resulting in a higher volume concentration of charges (i.e. excitation density/thickness) for the same excitation density has to be considered. For thinner layers, a population inversion can therefore already be achieved at lower excitation densities. It can be concluded that a minimal structure thickness for mode formation is needed combined with a rather small amount of perovskite for early population inversion to achieve low ASE thresholds.

As one can see from Figure 5a, the spectral shift of the ASE depends on the overall thickness of the structure. Moreover, in contrast to PL and absorption spectra, the spectral position of ASE shows a dependence not only on the layer thickness, but also on the total thickness of perovskite in the structure: In Figure 5a, it can be seen that ASE occurs at longer wavelengths for the MQW 20 (containing 100 nm of total perovskite thickness) than for the 50 nm‐thick bulk sample. This implies a larger gain due to more perovskite material. Hence, long‐wavelength photons can be amplified despite their low density of states.^[^ ^13^ ^]^ In contrast, the MQW 10 shows slightly blue‐shifted ASE compared to the bulk, even though both possess the same total perovskite thickness and the total structure of the former is thicker. Here the TPBi‐interlayers are playing an important role in lowering the gain for longer wavelengths.

We do not observe any significant optical performance degradation, even at high excitation intensities. However, the photostability of perovskite materials under optical excitation is, in general, much higher than for most organic materials.^[^ ^41^ ^]^

In conclusion, the development of coherence due to ASE or lasing is an extremely complicated process, which depends on many material parameters. We attribute these unusually low ASE thresholds observed in our MQWs to the exciton squeezing along the growth direction of the quantum well structures and all subsequent modifications to optical and structural properties of the whole system. These modifications have been proven through the linear absorption and PL emission measurements, and the XRR measurements, respectively. In particular, the exciton confinement has led to higher PL quantum efficiencies, which gives rise to easier population inversion and the earlier start of the stimulated emission. Furthermore, the decay dynamics in MQWs are considerably slower than in weakly confined (or bulk) systems, which further contributes to a longer exciton lifetime in its excited state and, consequently, to lower ASE threshold.

## 3 Summary and Outlook

In summary, we present a comprehensive study of the optical properties of CsPbBr_3_/TPBi MQWs with respect to the potential to utilize such a structure in perovskite‐based electrically pumped lasers. We accomplished strong increase in emission efficiency and could lower the threshold for ASE by using quantum well structures with high confinement. Quantum confinement is indicated by the observation of a blue‐shift in absorption and emission spectra with decreasing well width. Simulation of 1D MQWs having the same properties as the used materials showed very similar confinement energies. Since the effective carrier masses of TPBi are unknown, the simulation was fitted to the measurements by varying the effective masses. Here, a high degree of agreement with the measured values is reached for an effective electron mass of m* = 0.46 *m*_e_ and an effective hole mass of m* = 2.07 *m*_e_ for TPBi.

A large increase of emission intensity with thinner layers is observed with up to 15 times higher integrated emission intensity for a perovskite thickness of 3.2  nm compared to the bulk. Due to the lower absorbance, PLQY is even increased by 32 times. The increased overlapping of the charge carrier wave functions due to quantum confinement results in faster decay times and therefore, higher probability of radiative recombination. Furthermore, confinement prevents the dissolving of excitons, which also leads to higher emission intensities, and the high surface roughness may enhance the outcoupling efficiency.

Since vacuum processed perovskites are of interest for high carrier density applications such as lasers, ASE measurements were conducted. Here, very low thresholds down to 7.3 µJ cm^−2^ for a perovskite thickness of 8.7 nm are reached. Such low thresholds are unprecedented for CsPbBr_3_ thin‐film structures, up to now. With increasing perovskite layer thickness, an unusual increase of threshold is observed. This effect is described by the easier to achieve population inversion due to the lower layer thickness. The small perovskite thickness itself does not allow waveguide formation but for the whole structure, waveguide formation is possible, and therefore, the perovskite acts as layers of a gain medium within the propagation channel.

Due to the increased photoluminescence efficiency, the MQW structures presented here are excellent candidates for high‐efficiency perovskite‐based LEDs. The demonstration of successful ASE proves that MQW structures with CsPbBr_3_ are promising candidates for gain materials in future low‐energy lasing devices. The detailed role of waveguide formation in MQW structures has to be further analyzed to understand the observed thresholds, spectral positions, and intensities of ASE.

## Conflict of Interest

The authors declare no conflict of interest.

## Supporting information

Supporting Information

## Data Availability

The data that support the findings of this study are available from the corresponding author upon reasonable request.
