# Supplementary material for: Optical Properties of Perovskite‐Organic Multiple Quantum Wells
Source: Adv Sci (Weinh). 2022 Jul 3;9(24):2200379. doi: 10.1002/advs.202200379 (PMC9403629; doi:10.1002/advs.202200379)
Supplement: Supplementary file 1 — Supporting Information [file ADVS-9-2200379-s001.pdf]

ADVANCED SCIENCE
Open Access

Supporting Information

for *Adv. Sci.*, DOI 10.1002/advs.202200379

Optical Properties of Perovskite-Organic Multiple Quantum Wells

Tobias Antrack, Martin Kroll, Markas Sudzius, Changsoon Cho, Paulius Imbrasas, Miguel Albaladejo-Siguan, Johannes Benduhn, Lena Merten, Alexander Hinderhofer, Frank Schreiber, Sebastian Reineke, Yana Vaynzof and Karl Leo**

Supporting Material

1 Experimental

1.1 Sample Preparation

The perovskites and blocking layers are prepared by vacuum deposition using multi-source evaporation.
Before sample preparation, the substrates are cleaned in an ultra sonic bath for 10 minutes subsequently with soap water, distilled water, acetone, and isopropanol. Then, they are transferred into a vacuum deposition chamber (MINI PEROvap, CreaPhys GmbH, Germany). The vacuum deposition system has a cooled inner mantle to avoid re-evaporation and a temperature-stabilized substrate holder. The mantle temperature is set to −22 °C, the substrate holder is kept at room temperature, and evaporation is performed at a base pressure of 10^{-6} mbar. All materials are used as purchased from the supplier Sigma Aldrich. To produce $CsPbBr_3$, CsBr and $PbBr_2$ are evaporated simultaneously with rates of 0.1 Å/s and 0.115 Å/s, respectively, to ensure a stoichiometric ratio of 1 : 1.
To create the multiple-quantum well (MQW) structures, TPBi as blocking layer is first evaporated on the glass substrate, then $CsPbBr_3$ and TPBi are five times alternately evaporated on top, thus 5 perovskite quantum wells embedded in TPBi are created. TPBi is deposited with a rate of 0.3 Å/s. This production method allows to create very smooth thin films.

1.2 X-ray Characterization

Structural analysis is done by measuring X-ray diffraction (XRD) on a Bruker Discovery D8 system with a LYNXEYE_XE_T detector. Scans are typically performed from 10° to 45° with a step size of 0.02°, and the beam height is masked with a 0.2 mm slit to increase the angular resolution.
X-ray reflectivity (XRR) measurements are done in the same geometry as the XRD measurements, except that measurements are performed from 0° to 25° with a 0.1 mm-thick slit, and the rotatory absorber is set to automatic mode to compensate the intensity variations.

1.3 Optical Absorbance

For optical analysis, absorption spectra are obtained by measuring transmittance and reflectance spectra from 300 nm to 600 nm in an integrating sphere using a Shimadzu SolidSpec-3100. Absorbance is calculated in % for each wavelength via $A = 100\,\% - (T + R)$, where T and R are transmission and reflection in %, respectively.

1.4 Photoluminescence Quantum Yield

The photoluminescence quantum yield (PLQY) measurements are performed in an integrating sphere by using the three-measurements method [1]. An OBIS LS/LX CW-laser emitting at 405 nm is used as an excitation source and an Ocean Optics QW65 Pro as spectrometer. The excitation beam had an intensity of 20 mW and was focused to a spot diameter of 870 µm.

1.5 Amplified Spontaneous Emission

To measure amplified spontaneous emission (ASE), the samples are excited by a Ti:Sa femtosecond regenerative amplifier system (800 nm, frequency doubled to 400 nm) with a repetition rate of 5 kHz and a pulse length of 120 fs. We use a 5cm focal length lens to focus the pump beam. However, the sample is installed slightly off the focal plane of the lens. This mismatch is introduced intentionally to enlarge the pump beam's spot size to roughly 120 μm. In this way, we assure that the exciting area is sufficiently large to let the ASE develop reliably over the excited area with the lowest threshold possible [2]. We perform one calibration measurement for every experiment at relatively high pumping power, which is usually several times above the ASE threshold value and thus reliably measurable. For lower pump energies, we count on the linearity of our detector and proper calibration of ND filters, which we use to reduce pump beam intensity further. Our detector is a thermoelectrically cooled intensified CCD device, assuring linear response at the pump wavelength and visible spectral range within a large dynamic range. The pump intensity is varied by neutral density filters from 2.4 μJcm^{-2} to 198.8 μJcm^{-2} and the emitted photons are collected in direction of the pump beam behind the sample.

1.6 Scanning Electron Microscope

Cross sections of MQWs on glass were obtained with a Zeiss Gemini 500 SEM operated at 1.5 kV and observed with an Inlens detector under 10^{-5} mbar vacuum. To avoid samle charging, the films were sputtered with 15 nm of higly conductive Au:Pd alloy prior to imaging.

SEM Charge Accumulation Effects

In SEM measurements, there are often problems related to the accumulation of charge carriers. Electrons get trapped on electrically isolated spots or cannot flow away quickly enough. Therefore, they create a local electric field deflecting the electron beam right before reaching the sample surface, which results in image distortion [3]. Intense charge carrier accumulation effects are expected for the TPBi blocking layer and the grain boundaries since the conductivity is the lowest here. See Figure S1 for different distortion strengths induced by different integration times per pixel. To minimize such charging effects, the sample has to be made conductive such that the electrons can drift away from the illuminated spot. Therefore, a $15\,nm$ thick layer of gold was sputtered onto the sample (visible as the very bright top layer in all SEM images) to ensure high conductivity.

As a next step, the sample was broken by manual force (see Figure S2) to give a clear view of the cross-section. The process of breaking is very rapid and fast, which can lead to perovskite grains sticking out of the newly introduced surface. Thus, it can be assumed that we are not looking at a smooth surface but a very rough one. Outstanding perovskite grains would partly block the view to the organic layer or act as charge carrier accumulation points. Adding a gold layer on the cross-section face of the broken sample, however, results in no visible features at all. Therefore, charging effects are unavoidable.

All pictures are inevitably slightly blurry since we are at the resolution limit of our microscope, making thickness evaluations difficult. However, in some cases, it is still possible to get results that correspond well with the results obtained in the XRR measurements, which are expected to be much more precise. See Figure S3 for an example. Here, almost no squeezing of the overall structure was observed, which indicates an absence of charge carrier accumulation and, therefore, high conductivity through all layers. Such spots are very rare and were not found on all structures.

In some pictures, the top organic layer (directly facing the conducting gold layer) appears thicker than the other organic layers (Figure S4 as an example). Assuming that charge carrier accumulation is a considerable effect leading to distortion of single layers, this top organic layer should not be significantly affected by that since electrons are very likely to flow away through the gold contact. However, the top gold layer is often bent downwards or upwards as a result of the rapid breaking process, which can result in compressed or torn top organic layers.

Because of all these effects, our SEM measurements are not valid for thickness evaluation of the different layers, and we used only the XRR measurements to get the precise thickness values of the layers. Overall, we mainly took the cross-section SEM images to prove that compact and pinhole-free perovskite films were formed.

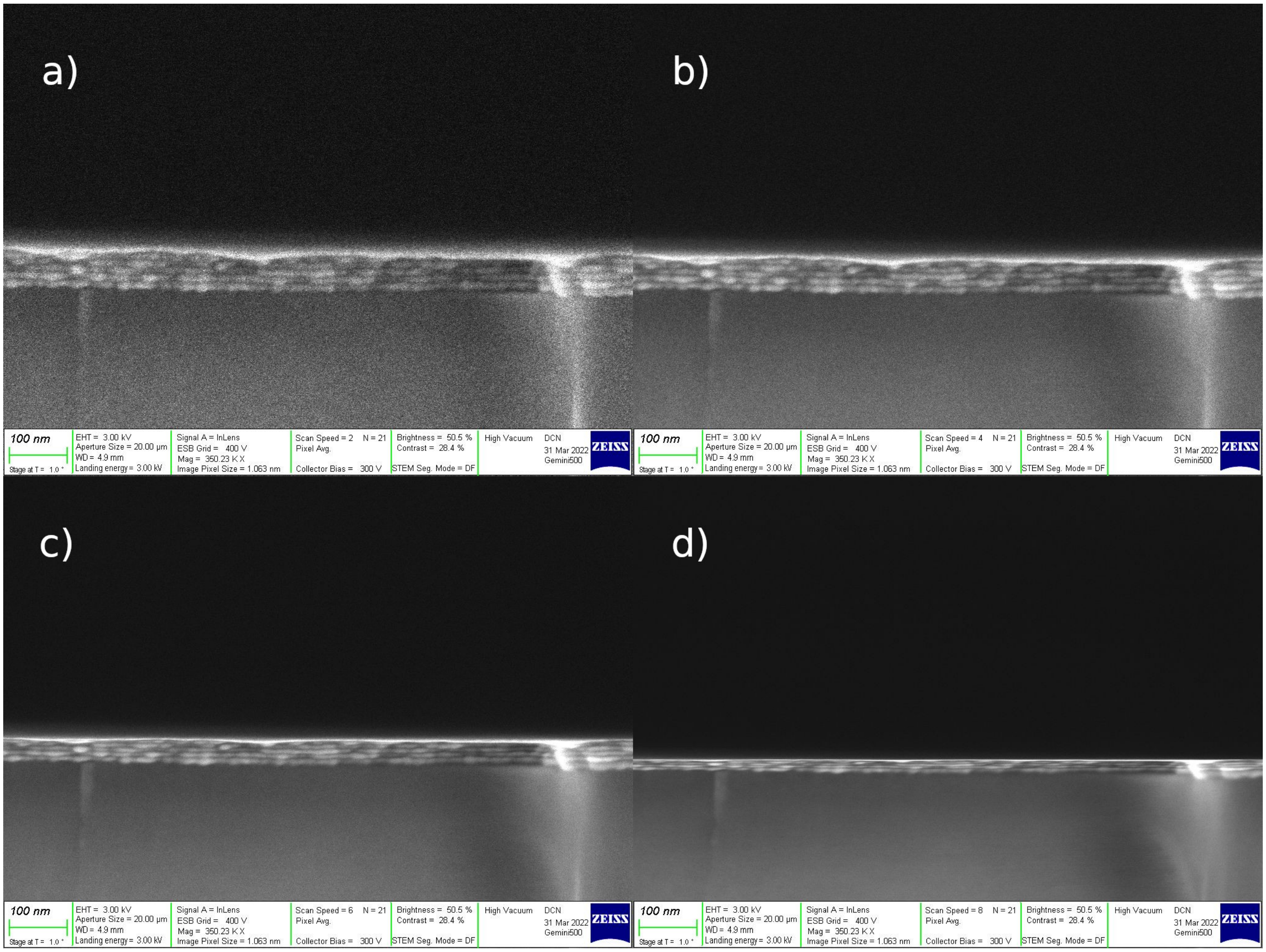

Figure S1: Demonstration of charging effects on a MQW 7 sample: SEM images of the same spot measured with different integration times per pixel (measurement parameter 'Scan Speed' varied from 2 to 8). With increasing integrating time, the whole image is vertically squeezed by more than a factor of 3 due to charge carrier accumulation.

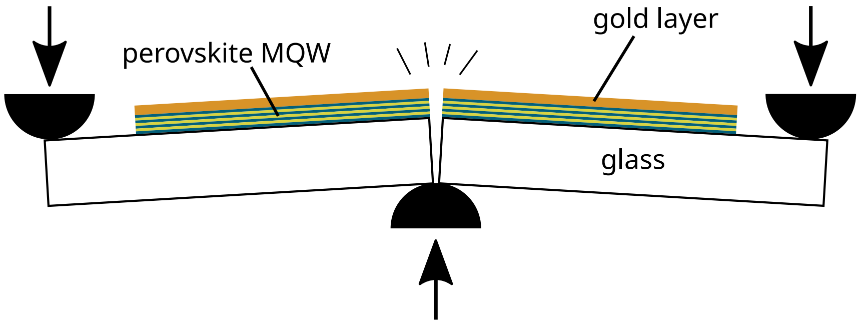

Figure S2: Schematic sketch of the process of sample breaking to obtain a cross-section area of the sample. This process is very rapid and the thin material layers get ruptured.

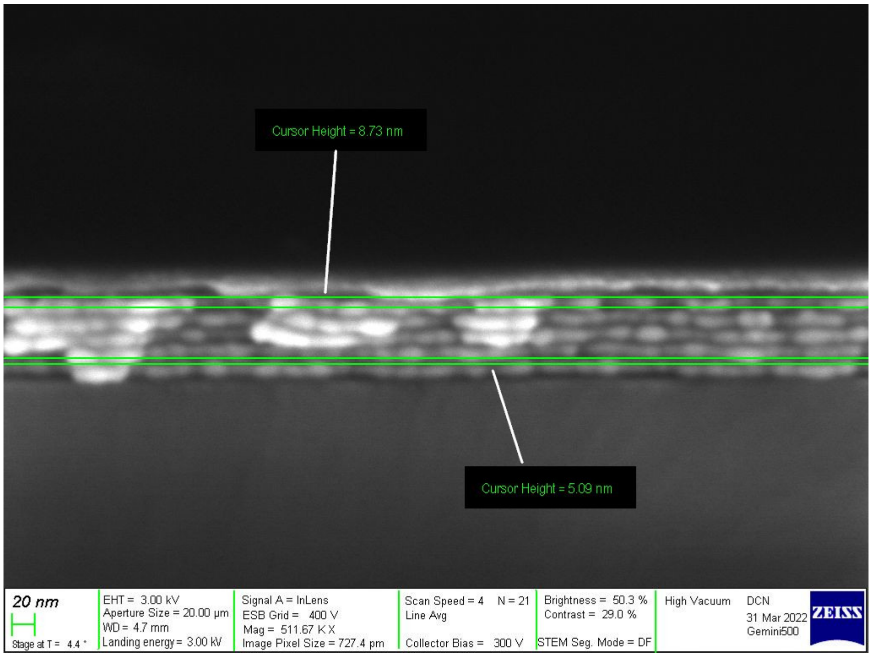

Figure S3: SEM image of a spot with almost no charging effect on a MQW 7 sample with a visual estimation of the thickness of a perovskite layer and an organic layer. The values fit very well with the obtained values by XRR (Table S1).

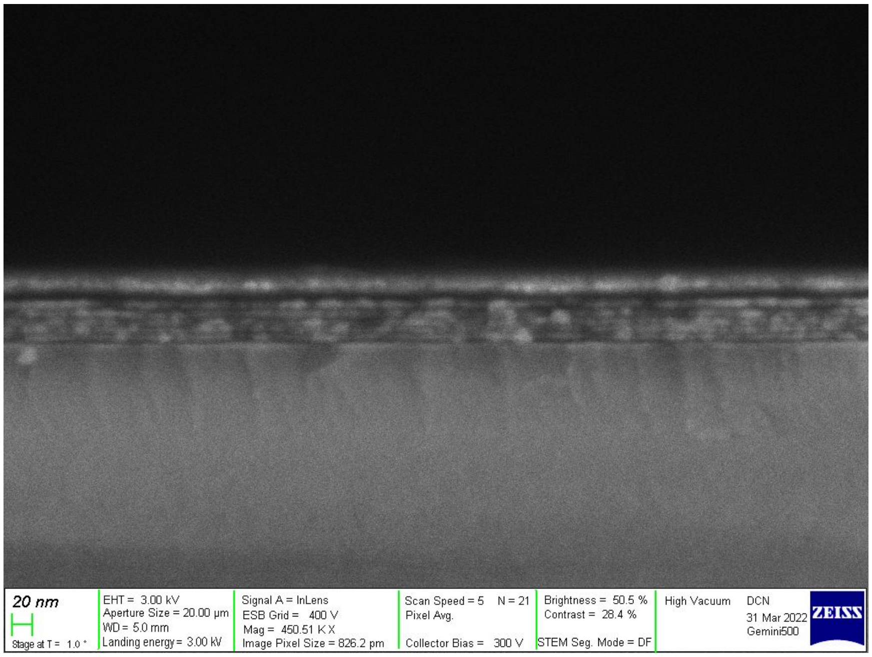

Figure S4: SEM image of a MQW 5 (5 nm thick TPBi layers and 5 nm thick $CsPbBr_3$ layers) sample. The very top bright layer is the gold layer that was sputtered on the sample prior to SEM measurement to ensure electrons can flow away from the area of exposure. The dark layer (TPBi) right underneath the gold layer appears to have a thickness very similar to the top $CsPbBr_3$ layer (next bright layer), which matches the design thicknesses. Due to the direct contact of the organic to the highly conductive gold layer, charge carriers can quickly transfer into the gold layer, and no charging effect in that region is expected.

2 Layer Thicknesses by XRR

Table S1: Measured perovskite and TPBi thicknesses and roughnesses for each structure. The results are obtained by fitting the XRR data assuming that all perovskite layers and all TPBi layers within a structure possess the same thicknesses.

Structure	MQW 3	MQW 5	MQW 7	MQW 10	MQW 20	bulk 50
$CsPbBr_3$ thickness / nm	3.2	5.5	8.7	10.0	20.5	50.4
$CsPbBr_3$ roughness / nm	3.6	4.8	4.3	1.5	2.6	2.4
TPBi thickness / nm	5.8	6.1	5.0	7.3	5.8	8.9
TPBi roughness / nm	1.1	1.9	0.9	1.1	1.8	1.3
Total perovskite / nm	16.0	27.5	43.5	50.0	102.5	50.4
Total structure / nm	50.8	64.1	73.5	93.8	137.3	68.2
% of perovskite in structure	31.5	42.9	59.2	53.3	74.7	73.9
Absolute log error / 10^{-1}	2.657	3.425	2.199	3.108	2.499	3.429

3 Tauc-Plot

To estimate the optical bandgap of a material from UV-VIS transmission data, a Tauc plot can be used. Here, $(\alpha h\nu)^n$ is plotted versus the photon energy $E_\nu = \frac{hc}{\lambda}$ with $\alpha = 2.303\,A/d$ being the absorption coefficient (determined via Beer-Lambert relation [4], $A = -log_{10}T$ [5] being absorbance, using transmission T and thickness d), h the Planck constant, ν the frequency of the photon and λ its wavelength. The exponent n denotes the nature of the assumed transition. Since $CsPbBr_3$ is expected to only have a direct bandgap [6], n is set to 1/2 [4]. By extrapolating both linear fits near the absorption edge of the experimental data versus incident photon energy, the optical bandgap can be estimated from the intercept of both fits (Figure S5) [7].

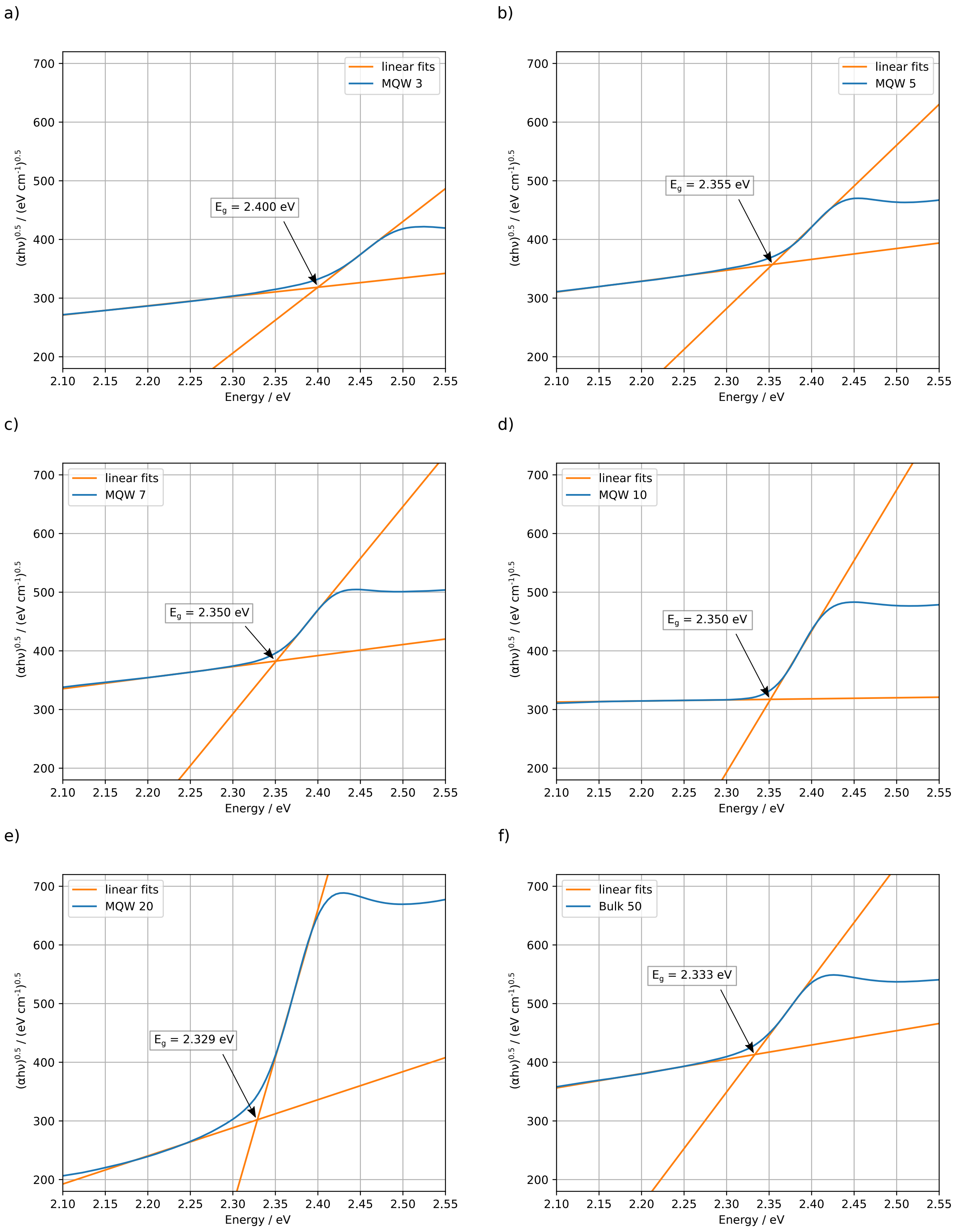

Figure S5: Tauc plots of all structures.

4 Confinement Simulation

The well material $CsPbBr_3$ has an optical band gap in bulk of 2.30 eV while TPBi possesses 3.26 eV [8]. Combining both materials forms a type I quantum well with a well depth of 0.41 eV for electrons and 0.55 eV for holes [8].

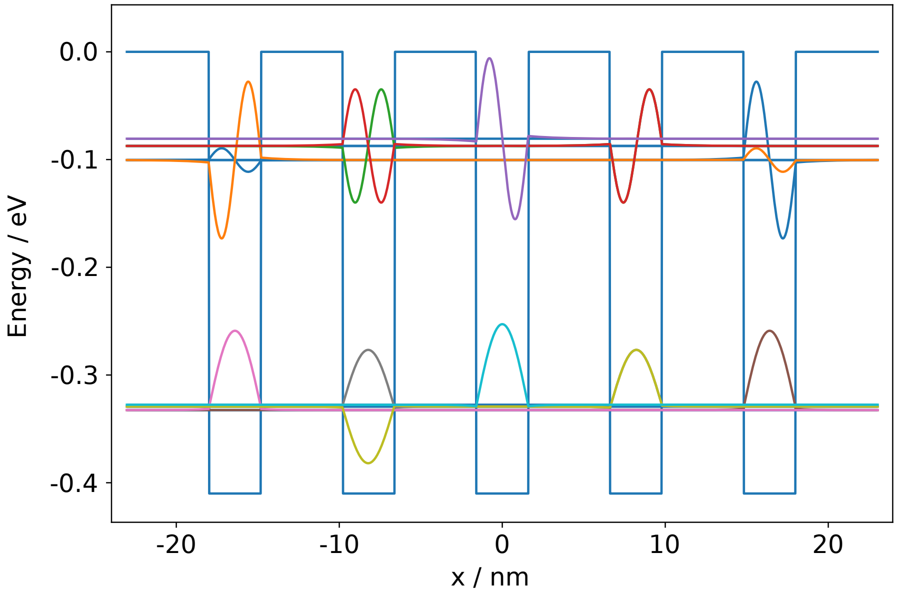

Figure S6: Step-like potential with the energy eigenstates shown as blue horizontal lines and their corresponding wave functions with arbitrary amplitude for a well width of 3.2 nm. The formation of subbands due to weak inter-well coupling of the wave functions is clearly visible. For this simulation, the potential is divided into 2000 finite steps.

5 Decay Properties

After exciting the sample with a laser diode (PicoQuant LDH-D-C-375) at 375 nm with pulse width of 44 ps, the emission is collected from a photomultiplier tube (PicoQuant PMA Hybrid) and data acquisition is handled by a TCSPC module (PicoQuant TimeHarp 260).
To characterize the decay traces, fitting is conducted assuming two exponential decay components and a power-law decay:

$$I(t) = A_1 \cdot \exp\left(\frac{-t}{\tau_1}\right) + A_2 \cdot \exp\left(\frac{-t}{\tau_2}\right) + A_3 \cdot \frac{1}{(1+\frac{t}{\tau_3})^P} \tag{1}$$

where A, τ, and t are the intensity, decay constant, and time for each component, respectively, and P the exponent of the power-law. All fitted parameters are listed in Table S2.
Recombinations of bound and free excitons typically result in exponential decay traces [9], whereas bimolecular decay mechanisms are expected to follow power-law decay traces [10].

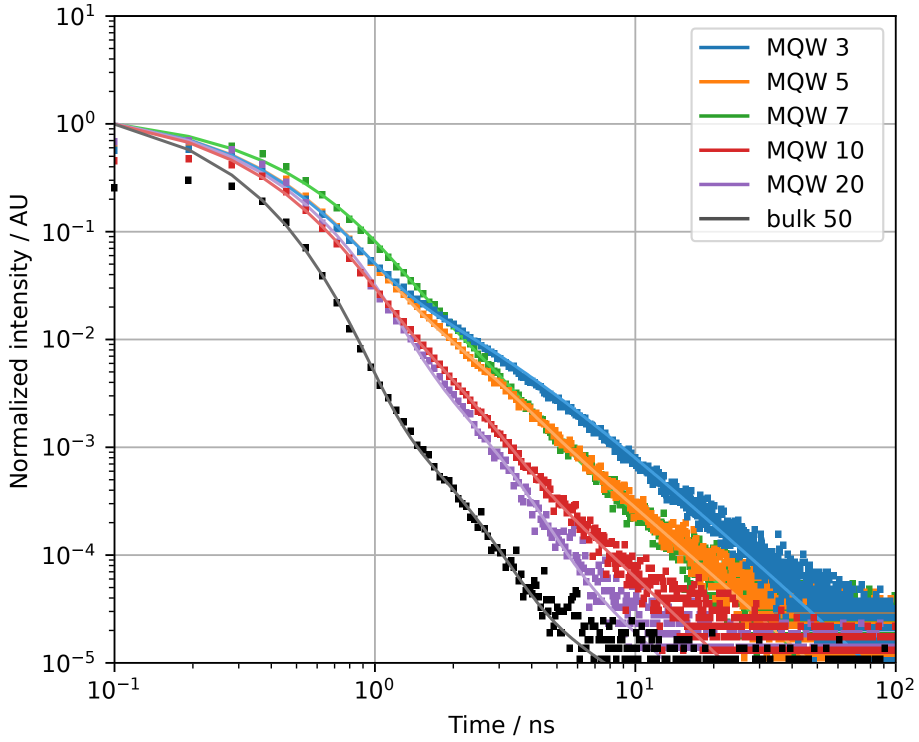

Figure S7: Emission decay traces of the different layer structures.

Table S2: Fitting parameters of the decay traces in Figure S7 using equation 1. Two exponential decay mechanisms ('exp 1', 'exp 2') are considered where 'exp avg' denotes the average decay time of both exponential functions weighted with their intensities. An additional power-law decay is added to the fit function where 'power-law' denotes the decay constant and 'exponent' the exponent of the decay function.

Parameter	MQW 3	MQW 5	MQW 7	MQW 10	MQW 20	bulk 50
A_1 / AU	2.223	1.905	1.617	2.583	1.849	5.646
τ_1 / ns	0.278	0.291	0.372	0.256	0.291	0.183
A_2 / AU	0.021	0.029	0.059	0.066	0.021	0.019
τ_2 / ns	0.397	1.232	1.227	0.868	1.144	0.834
$\tau_{\text{avg, exp}}$ / ns	0.279	0.348	0.464	0.305	0.327	0.193
A_3 / AU	0.172	0.373	0.319	0.467	0.305	0.019
τ_3 / ns	1.486	0.515	0.474	0.328	0.169	0.184
$P/1$	2.09	2.029	1.99	2.18	2.117	1.564

6 Amplified Spontaneous Emission: Threshold Determination

To characterize ASE properties, the ASE signal has to be distinguished from the much broader PL signal. Here, the ratio of ASE to PL peak height $R_{\mathrm{ASE/PL}}$ is obtained by:

$$R_{\mathrm{ASE/PL}} = \frac{I_{\mathrm{ASE}}}{I_{\mathrm{PL}}} \tag{2}$$

where I_{ASE} is the maximum spectral intensity within the ASE range, and I_{PL} is the spectral intensity at the PL peak wavelength (obtained by fitting a Gaussian function to the peak). Both values were averaged over the next 3 measurement points (resolution of 16 measurement points per spectral nm) in the spectra to minimize noise effects. Since the peak position of the ASE emission is shifting in dependence of the pumping intensity, a spectral range for the ASE signal is manually set. The left boundary of the spectral ASE range is set as the wavelength where the additional ASE peak arises from the PL signal, whereas for the right boundary the ASE peak position at maximum excitation intensity is chosen. The ASE peak position and the intensity were detected by software for each pumping intensity.
The threshold value for the ASE was determined by fitting a linear function in the linear PL regime before the ASE takes place, i.e., low pumping power, and a second linear function after in the ASE regime, i.e., high pumping power, in a double logarithmic presentation of $R_{\mathrm{ASE/PL}}$ versus pumping intensity (Figure S8). Afterwards, the ASE threshold value was obtained from the intercept of both functions.

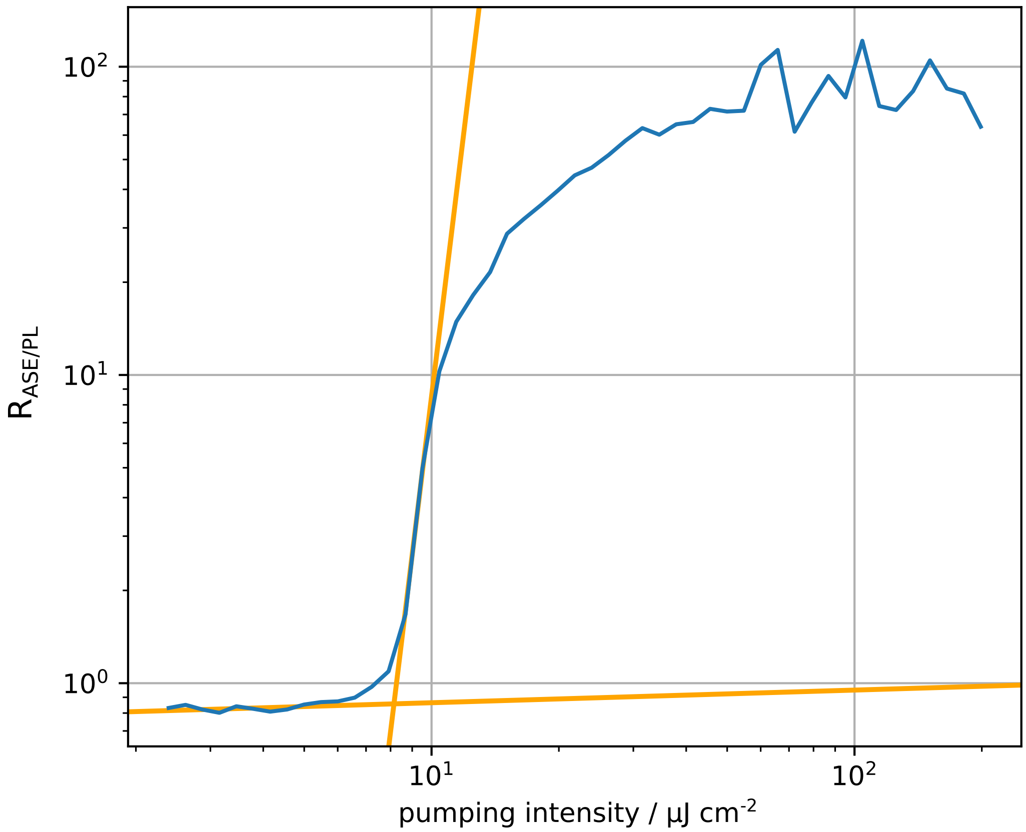

Figure S8: Determination of ASE threshold for the 50 nm bulk sample. A double logarithmic representation shows the measured ratio of ASE to PL signal height ($R_{\mathrm{ASE/PL}}$) versus the pumping power. The two linear fits intercept at a pumping intensity of 8.2 µJ/cm^2. The maximum values of the PL spectrum were evaluated at 514.8 nm. The maximum spectral intensity was obtained for the ASE signal in the spectral range of 530-550 nm.

References

[1] J. C. de Mello, H. F. Wittmann, and R. H. Friend. "An improved experimental determination of external photoluminescence quantum efficiency". In: *Advanced Materials* 9.(3) (1997), pp. 230–232.

[2] C. Cho, A. Palatnik, M. Sudzius, R. Grodofzig, F. Nehm, and K. Leo. "Controlling and Optimizing Amplified Spontaneous Emission in Perovskites". In: *ACS Applied Materials & Interfaces* 12.(31) (2020), pp. 35242–35249. ISSN: 1944-8244. DOI: 10.1021/acsami.0c08870.

[3] M. P. Davidson and N. T. Sullivan. "Investigation of the effects of charging in SEM-based CD metrology". In: *Metrology, Inspection, and Process Control for Microlithography XI*. Vol. 3050. International Society for Optics and Photonics. 1997, pp. 226–242.

[4] M. Shkir, V. Ganesh, I. S. Yahia, and S. AlFaify. "Microwave-synthesis of La3+ doped PbI2 nanosheets (NSs) and their characterizations for optoelectronic applications". In: *Journal of Materials Science: Materials in Electronics* 29.(18) (2018), pp. 15838–15846. ISSN: 1573-482X. DOI: 10.1007/s10854-018-9670-3.

[5] J. Gordon and S. Harman. "A graduated cylinder colorimeter: An investigation of path length and the Beer-Lambert law". In: *Journal of chemical education* 79.(5) (2002), p. 611.

[6] L. Schmidt-Mende, V. Dyakonov, S. Olthof, F. Ünlü, K. M. T. Lê, S. Mathur, A. D. Karabanov, D. C. Lupascu, L. M. Herz, and A. e. a. Hinderhofer. "Roadmap on organic–inorganic hybrid perovskite semiconductors and devices". In: *APL Materials* 9.(10) (2021), p. 109202. DOI: https://doi.org/10.1063/5.0047616.

[7] P. S. Shinde, G. H. Go, and W. J. Lee. "Facile growth of hierarchical hematite (α-Fe 2 O 3) nanopetals on FTO by pulse reverse electrodeposition for photoelectrochemical water splitting". In: *Journal of Materials Chemistry* 22.(21) (2012), pp. 10469–10471.

[8] K. J. Lee, N. A. Merdad, P. Maity, J. K. El-Demellawi, Z. Lui, L. Sinatra, A. A. Zhumekenov, M. N. Hedhili, J.-W. Min, J.-H. Min, L. Gutiérrez-Arzaluz, D. H. Anjum, N. Wei, B. S. Ooi, H. N. Alshareef, O. F. Mohammed, and O. M. Bakr. "Engineering Band-Type Alignment in CsPbBr3 Perovskite-Based Artificial Multiple Quantum Wells". In: *Advanced Materials* 33.(17) (2021), p. 2005166. DOI: https://doi.org/10.1002/adma.202005166.

[9] G. E. Bunea, W. D. Herzog, M. S. Ünlü, B. B. Goldberg, and R. J. Molnar. "Time-resolved photoluminescence studies of free and donor-bound exciton in GaN grown by hydride vapor phase epitaxy". In: *Applied Physics Letters* 75.(6) (1999), pp. 838–840. DOI: 10.1063/1.124530.

[10] C.-K. Sun, S. Keller, G. Wang, M. S. Minsky, J. E. Bowers, and S. P. DenBaars. "Radiative recombination lifetime measurements of InGaN single quantum well". In: *Applied Physics Letters* 69.(13) (1996), pp. 1936–1938. DOI: 10.1063/1.117627.
